# Structural Basis of Regioselective Bromination of Tricyclic Tryptoline by the Tryptophan Halogenase Thal

**DOI:** 10.1002/cbic.202500246

**Published:** 2025-06-17

**Authors:** Simon Bork, Caroline Besse, Norbert Sewald, Hartmut H. Niemann

**Affiliations:** ^1^ Department of Chemistry Structural Biochemistry Bielefeld University Universitätsstraße 25 33615 Bielefeld Germany; ^2^ Department of Chemistry Organic and Bioorganic Chemistry Bielefeld University Universitätsstraße 25 33615 Bielefeld Germany

**Keywords:** enzymatic halogenation, enzyme catalysis, indole alkaloids, protein structures, regioselectivity

## Abstract

Flavin‐dependent halogenases (FDHs) carry out substrate‐specific and regioselective halogenation reactions in the biosynthesis of various halogenated natural compounds. Several FDHs convert non‐native substrates in vitro. However, obtaining experimental structures of FDHs with non‐native substrates remains challenging, and docking often produces ambiguous results. Hence, there is a lack of data on how non‐native substrates bind to FDHs. Here, we show that the tryptophan 6‐halogenase Thal efficiently brominates the tricyclic indole derivative tryptoline (1,2,3,4‐tetrahydro‐β‐carboline) with high regioselectivity. The two point mutations G113S and G469S improve regioselectivity even further. A crystal structure reveals how tryptoline binds to the active site of Thal. The halogenated carbons are located close to the catalytic lysine, and the NH of tryptoline's tetrahydropyridine is positioned like the amino group of the native substrate tryptophan. The substrate binding loop of Thal is closed, again resembling the binding of tryptophan. Our work extends the range of non‐native substrates accepted by Thal, confirming the versatility of this FDH. Moreover, it is a rare example of an FDH structure in complex with a non‐native substrate.

## Graphical abstract

This study shows that the flavin‐dependent tryptophan halogenase Thal also regioselectively halogenates the tricyclic non‐native substrate tryptoline, a β‐carboline with various pharmacological activities. Interestingly, Thal brominates a different position of the shared pharmacophore indole in tryptoline than it does in tryptophan. A crystal structure of Thal in complex with tryptoline explains this different regioselectivity.

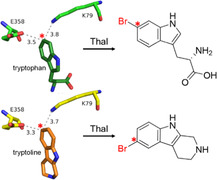

## 1 Introduction

Halogenated compounds are essential building blocks for a variety of pharmaceuticals and agrochemicals.^[^ ^1^, ^2^, ^3^, ^4^ ^]^ The necessary halogenation of these molecules by chemical synthesis requires harsh conditions and lacks regioselectivity.^[^ ^5^ ^]^ FDHs are a promising alternative since they are able to halogenate different moieties under mild conditions with high regioselectivity.^[^ ^6^, ^7^ ^]^ Most of the well‐studied FDHs natively incorporate the halogen substituent into smaller molecules like pyrrole, phenol, indole, or tryptophan.^[^ ^6^, ^8^ ^]^ In many cases, the desired halogenated building blocks are bigger molecules and the halogenation of these with known enzymes remains challenging. Therefore, several attempts were made to increase the substrate scope of FDHs,^[^ ^9^, ^10^ ^]^ and remarkable results have recently even been achieved in the halogenation of tryptophan within peptides and proteins.^[^ ^11^, ^12^, ^13^, ^14^ ^]^

For the tryptophan 7‐halogenase RebH, Lewis and coworkers used a substrate walking approach to increase the size of accepted substrates and achieved, for example, a ≈40‐fold increase in conversion of the sterically demanding pentacyclic indole alkaloid yohimbine after only three rounds of directed evolution.^[^ ^10^ ^]^ Another variant, namely, RebH 3‐SS, showed an almost 70‐fold increased conversion of tricyclic tryptoline. Tryptoline belongs to the group of β‐carbolines, a family of natural and synthetic indole‐containing compounds with broad biological activities, like anticancer, neuropharmacological, or antimalarial.^[^ ^15^, ^16^, ^17^ ^]^ Tryptoline can, for example, bind imidazoline receptors and inhibit monoamine oxidase A.^[^ ^18^, ^19^ ^]^

Three of the six mutations of RebH 3‐SS (N467T, G112S, and N470S) are located in the substrate binding site. Comparing the substrate binding sites of RebH 3‐SS and the tryptophan 6‐halogenase Thal, only two amino acids are different. Like wild‐type RebH, Thal has a glycine at position 113 (equivalent to position 112 in RebH), while RebH 3‐SS has a serine at position 112. Both RebH and RebH 3‐SS have a serine at position 469, whereas Thal has a glycine. Two of the mutations in RebH 3‐SS, N467T and N470S, are the native amino acids at homologous positions of Thal.^[^ ^10^, ^20^, ^21^ ^]^

The similarity of the active sites of RebH 3‐SS and Thal caught our attention because we had previously obtained structures of Thal with two non‐native substrates, while generally experimental structures of FDHs with non‐native substrates are still extremely scarce. Here, we analyzed whether Thal and its variants Thal G113S and Thal G469S are able to halogenate tryptoline. We determined the crystal structure of Thal in complex with tryptoline to gain insights into how Thal binds bigger substrates. This structure explains the experimentally observed regioselectivity of tryptoline bromination by Thal.

## 2 Results and Discussion

### 2.1 Biocatalytic Halogenation of Tryptoline

**Scheme 1:**
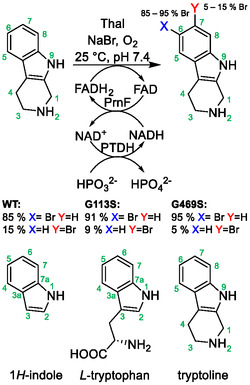
Thal‐catalyzed halogenation of tryptoline, including the cofactor regeneration system. PrnF: flavin:NADH reductase; PTDH: phosphite dehydrogenase.

To characterize the biocatalytic halogenation of tryptoline by FDHs, we determined the conversion of tryptoline by Thal and its variants Thal G113S and Thal G469S, as well as by RebH and its variants RebH N470S and RebH G112S N470S (RebH 2S). A sequence alignment and structural alignments of residues involved in substrate binding are shown in Figures S1 and S2, Supporting Information. As FDHs need FADH_2_ as a cofactor, the flavin:NADH reductase PrnF and a phosphite dehydrogenase (PTDH) were utilized to regenerate FADH_2_ and NADH, respectively, with phosphite as a sacrificial substrate (**Scheme** **1**). Using the halogenase and the cofactor regeneration system, halogenation can proceed with phosphite, NaBr, and oxygen as substrates along with only catalytic amounts of FAD and NAD. It should be noted that the numbering of the indole moiety in tryptoline differs from that of indole and tryptophan (Scheme 1). This needs to be considered when comparing the regioselectivities toward the substrates.

**Figure 1:**
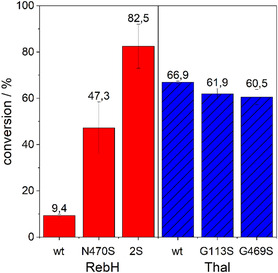
Conversion of tryptoline by RebH and Thal variants (10 μM) after 3 h. Conversion was determined via RP‐HPLC with n = 3 ± SD.

We first assessed the bromination of tryptoline by wild‐type RebH and two RebH variants. The N470S mutation in RebH led to a 5‐fold increased conversion compared to the wild type. The combination of the mutations N470S and G112S (2S) led to a further increase to almost 9‐fold conversion compared to the wild type (**Figure** **1**). Wild‐type Thal showed good conversion, which is higher than the conversion of RebH N470S. The similar conversion is not surprising since the N470S mutation in RebH increases the similarity of the active sites of RebH and Thal. Incorporation of the mutations G113S and G469S into Thal did not result in increased conversion (Figure 1), despite the variants having higher similarity to RebH 2S, which achieved the best conversion. Especially for Thal G113S, a higher conversion was expected, as the homologous mutation in RebH led to an increased conversion. However, the effect of individual mutations on enzyme activity and regioselectivity is well known to depend on the sequence context. In RebH, active site mutations that have a large effect on site selectivity in an evolved variant led to very low, if any, activity in wt RebH.^[^ ^22^ ^]^ Given a sequence identity of 64% between Thal and RebH, it may not be surprising that the same mutation shows different effects in the two proteins.

Thal G113S and Thal G469S yielded conversions similar to the wild type but achieved better regioselectivity. Conversion of tryptoline to bromotryptoline on a bigger scale and analysis of the bromination sites by nuclear magnetic resonance (NMR) (Figures S3–S10, Supporting Information) showed that Thal produced around 85% 6‐bromotryptoline and 15%7‐bromotryptoline. The halogenation position in tryptoline differs from that of the native substrate. The major product 6‐bromotryptoline is structurally equivalent to 5‐bromotryptophan, and the minor product 7‐bromotryptoline is equivalent to the native product 6‐bromotryptophan. For Thal G113S only 10% and for Thal G469S only 5% of the total product was 7‐bromotryptoline (Scheme 1). This is consistent with the results for RebH. While the wild type produces an approximate 1:1 mixture of 6‐ and 7‐chlorotryptoline,^[^ ^10^ ^]^ the variant RebH N470S produces only ≈5% of 7‐bromotryptoline (Figure S11, Supporting Information). With the RebH 2S double mutant, only traces of 7‐bromotryptoline are present in the product (Figure S12, Supporting Information). As the mutations G113S and G469S further approximate the active sites of Thal to that of RebH 2S, the increase in regioselectivity in Thal is comparable to that caused by the mutations in RebH.

The kinetic parameters of Thal for tryptoline (Figures S13 and S14, Supporting Information) were significantly worse (20‐fold for *K*_M_ and *k*_cat_) compared to the natural substrate l‐tryptophan (Figures S15 and S16, Supporting Information), which is consistent with the structural variation (**Table** **1**). A similar or rather slightly higher discrepancy was previously described for RebH (Table 1). A comparison of the tryptoline parameters of Thal with those of RebH, determined by Payne et al.,^[^ ^10^ ^]^ shows a similar *K*_M_ value, while the *k*_cat_ value is 2.5‐ to 5‐fold higher for Thal, which is also consistent with the better conversions by Thal. It is important to note that in this work the parameters for bromination were studied, while Payne *et al.* determined the parameters for chlorination.^[^ ^10^, ^23^ ^]^

**Table 1:** Kinetic data for bromination of tryptophan and tryptoline by Thal and for chlorination of tryptophan and tryptoline by RebH published by Payne et al.^[^ ^10^, ^23^ ^]^

	substrate	[*k*_cat_/min^−1^]	[*K*_M_/μM]	[*k*_cat_/*K*_M_/min^−1^μM^−1^]
Thal	l‐Trp	1.53 ± 0.06	10.6 ± 1.6	0.144
	tryptoline	0.066 ± 0.004	199 ± 32	3.0 × 10^−4^
RebH	l‐Trp^[^ ^23^ ^]^	1.1	7,3	0.150
	tryptoline^[^ ^10^, ^23^ ^]^	0.027	216	1.25 × 10^−4^
		0.013	144	9.05 × 10^−5^

### 2.2 Crystal Structure of Thal in Complex with Tryptoline

**Figure 2:**
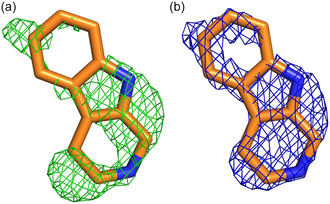
a) mF_o_‐DF_c_ (3 σ) density (green mesh) of tryptoline (orange carbon atoms) before placing the ligand and b) 2mF_o_‐DF_c_ (1 σ) density (blue mesh) of the final model.

To gain a better understanding of how Thal achieves regioselective halogenation of non‐native substrates and how the structures can be altered to allow for halogenation of similar or even bulkier substrates, we aimed at solving the structure of Thal in complex with tryptoline by soaking crystals of Thal with a tryptoline‐containing buffer. The structure was solved to a resolution of 2.2 Å, and it revealed the typical crystal packing with two chains per asymmetric unit (data collection and refinement statistics in Table S2, Supporting Information). The tryptophan binding site of chain A contained a large and almost planar difference density, while the active site of chain B appeared to be empty (Figure S17, Supporting Information). Like in other structures of Thal, the substrate binding loop of chain B is disordered (residues 451–460) due to no ligand being present.^[^ ^24^, ^25^ ^]^ After several cycles of refinement and model building, the difference density was interpretable as tryptoline in a unique binding pose (**Figure** **2**a). The placed and refined ligand has an occupancy of 67% and is almost completely enveloped in *2mF*_*o*_ *‐DF*_*c*_ (1 σ) density (Figure 2b).

To scrutinize the correct binding pose, we refined the structure with four potentially reasonable binding poses of tryptoline (Figure S18, Supporting Information). After ten cycles of refinement using simulated annealing, the binding pose we settled on (Figure S18a, Supporting Information) was the only one, which resulted in a good representation of the given electron density and reasonable polar contacts. Furthermore, a polder map^[^ ^26^ ^]^ with tryptoline as the omitted molecule did not reveal additional putative binding poses of tryptoline in our crystal structure (Figure S19, Supporting Information). In addition to the tryptoline in the active site of chain A, both chains showed fortuitous binding of tryptoline at a position where physiologically ring I of the FAD isoalloxazine moiety binds. Chain A additionally binds a second tryptoline in the flavin adenine dinucleotide (FAD) binding region. Nevertheless, the FAD binding loop (amino acids 38–45) adopts an open conformation.

**Figure 3:**
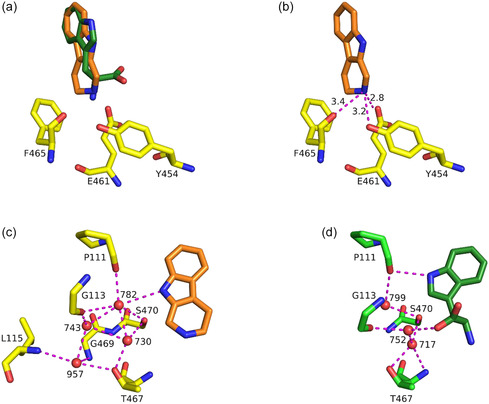
Orientation of tryptoline in Thal. a) Binding pose of tryptoline (orange carbon atoms) compared to L‐Trp (dark‐green carbon atoms, from PDB: 6h44). b) Polar contacts (magenta dashed line, distances in Å) of tryptoline (orange carbon atoms) to surrounding amino acids (yellow carbon atoms). c,d) Water network coordinating tryptoline (orange carbon atoms) and L‐Trp (dark‐green carbon atoms, PDB: 6h44).

Tryptoline binds in a similar way as the natural substrate l‐Trp (PDB: 6h44),^[^ ^21^ ^]^ with the aromatic moiety in the same plane but slightly rotated (**Figure** **3**a). The position of the nitrogen of tryptoline's tetrahydropyridine moiety (N2) and the amino group of the tryptophan is almost congruent.

**Figure 4:**
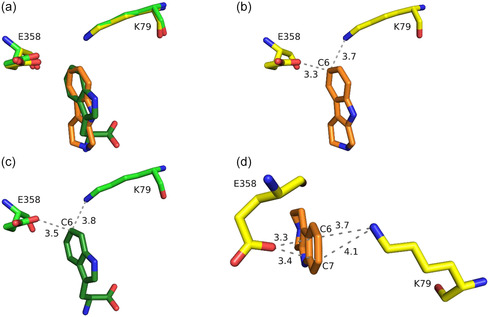
a) Position of tryptoline (orange carbon atoms) relative to catalytic amino acids K79 and E358 (yellow carbon atoms) compared to L‐Trp (dark‐green and green carbon atoms, PDB: 6h44). b) Distances (in Å) of C6 of tryptoline to K79 and E358. c) Distances (in Å) of C6 of L‐Trp to K79 and E358. d) Comparison of the C6 and C7 distances (in Å) of tryptoline to K79 and E358.

This leads to the same polar contacts of tryptoline's N2 to the side chains of Y454 and E461 and the backbone carbonyl of F465 as described for tryptophan's amino group (Figure 3b).^[^ ^21^ ^]^ We assume that these interactions are crucial to bind similar substrates in a defined pose to ensure regioselective halogenation. Additionally, the interactions with surrounding aromatic amino acids (H110, F112, F465, W466) might be crucial to stabilize the binding position and orientation of tryptoline as described for l‐Trp.^[^ ^21^ ^]^ To ensure regioselectivity, two‐component halogenases position their substrate in a way that the halogenated carbon atom has the shortest distance to the εNH_3_^+^ group of the catalytic lysine.^[^ ^21^, ^27^, ^28^ ^]^ This also applies to the non‐native substrate tryptoline in Thal. When bound to Thal, the catalytically preferred C6 of tryptoline is sterically almost congruent with the C6 of l–Trp (**Figure** **4**a) and has the shortest distance toward the catalytic amino acids K79 and E358. The distances of 3.7 Å to K79 and 3.3 Å to E358 (Figure 4b) are very similar to the distances of tryptophan's C6 with 3.8 and 3.5 Å, respectively (Figure 4c). In contrast to the natural substrate l‐Trp, conversion of tryptoline is not completely regioselective with ≈85% 6‐bromotryptoline and ≈15% 7‐bromotryptoline. A reason for the formation of the second regioisomer could be the comparable distances of tryptoline's C6 and C7 to K79 (3.7 Å for C6 and 4.1 Å for C7) and E358 (3.3 Å for C6 and 3.4 Å for C7) (Figure 4d). The similar distances of C7 might lead to a less favored, but still occurring, halogenation of this position. For tryptophan, the distances of C7 (which corresponds to C8 of tryptoline but is sterically closest to C7 of tryptoline when bound to Thal) are 4.6 Å (to K79) and 3.9 Å (to E358), which may be sufficient to prevent halogenation of this position (Figure S20, Supporting Information). Another reason might be that tryptoline adopts a low occupied second binding pose in a slightly rotated or even flipped orientation that would position its C7 closer to K79. The mutations G113S and G469S both improved regioselectivity compared to wild‐type RebH. Both of them might prevent the tryptoline from binding in this putative less favored binding pose by altering the water structure involved in coordinating the ligand, as observed for the natural substrate l–Trp in Thal^[^ ^21^ ^]^ (Figure 3c,d). As stated before, a polder map, which is intended to show the electron density of low‐occupancy ligands more clearly, provided no hint for a second binding pose. However, even polder maps will not show density for ligands with very low occupancy. Thus, we cannot exclude with certainty the existence of a second binding pose with very low occupancy.

**Figure 5:**
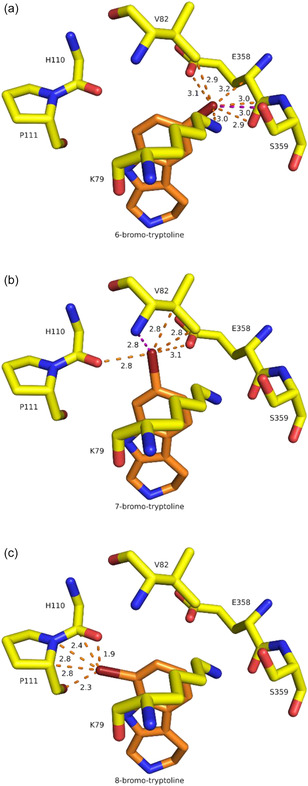
Steric conflicts of potential halogenation products of tryptoline. Tryptoline is replaced by 6‐, 7‐, and 8‐bromotryptoline (orange carbon atoms). Clashes are shown as orange dotted lines, and polar contacts as magenta dotted lines. All lengths are given in Å. Sidechain of H110 not displayed.

Additionally, steric conflicts of the potential halogenation products might play a role here as discussed by Schnepel et al. for the halogenation of a dipeptide by Thal.^[^ ^11^ ^]^ To test this hypothesis, we replaced tryptoline *in silico* by 6‐, 7‐, and 8‐bromotryptoline (**Figure** **5**) using the guided ligand replacement tool^[^ ^29^ ^]^ in *phenix*. Several experimental structures of halogenases in complex with the substrate and halogenated product (e.g., 7‐Cl‐Trp in PrnA, 6‐Cl‐Trp and BorH and two differently chlorinated malbrancheamides in MalA’) showed very little displacement of the halogenated product compared to the substrate.^[^ ^28^, ^30^, ^31^ ^]^ Therefore, a direct replacement of tryptoline with probable halogenation products should result in plausible binding scenarios. Replacing tryptoline in Thal leads to several severe clashes of the bromine substituent in 8‐bromotryptoline and could further explain why we observed no formation of the C8 product during biocatalysis. For both the C6 and C7 products, no major steric conflicts occur, with those at C7 being more severe (shorter distances) than at C6. Hence, there should be enough space at both positions to form the corresponding product. Based on the steric conflicts, we could not conclude whether halogenation at C6 would be preferred over C7.

Compared to RebH, Thal shows higher conversion and higher *k*_cat_/*K*_M_ toward tryptoline. One reason for this could be that Thal provides more space in its active site than RebH, which could make Thal a better candidate to adjust its active site to the halogenation of bigger substrates.^[^ ^21^ ^]^ The mutation N470S in RebH leads in the single mutant presented in this work (RebH N470S) and in RebH 3–SS from Lewis and coworkers^[^ ^10^ ^]^ to an increased conversion of tryptoline comparable to that of Thal. A plausible explanation is that the corresponding serine (S470) is already present in Thal. Based on structure‐guided mutagenesis, Micklefield and coworkers were able to alter the substrate scope of PrnA to non‐indolic substrates like kynurenine, anthranilamide, and different anilines by mutating E450 and F454 in PrnA.^[^ ^9^ ^]^ The corresponding amino acids E461 and F465 in Thal are both involved in positioning tryptoline via polar contacts and in the case of F465 additionally via interactions with its side chain (Figure 3b). These positions are promising candidates to further alter the substrate scope and increase the size of convertible substrates by Thal or related halogenases.

The literature contains several unsuccessful attempts to solve structures of FDHs in complex with native or non‐native substrates. The Trp 5‐halogenase AbeH and the related Trp 6‐halogenase Tar14 were co‐crystallized or soaked with their native substrate, but there was no electron density for bound Trp.^[^ ^31^, ^32^ ^]^ Likewise, soaking crystals of CtcP with its native substrate tetracycline did not result in a complex structure.^[^ ^33^ ^]^ Several attempts to obtain structures of RebH variants with non‐native substrates were not successful, e.g., for RebH‐F454K with anthranilic acid and for RebH 8 F and 10S with tryptamine.^[^ ^9^, ^22^ ^]^ Many attempts to crystallize VirX1 in complex with various substrates did not yield sufficiently diffracting crystals for structural analysis.^[^ ^34^ ^]^ Crystals of PltM were soaked with the native substrate phloroglucinol and six non‐native substrates, but only phloroglucinol yielded a difference in electron density and resulted in a complex structure.^[^ ^35^ ^]^

There may be various reasons for the challenges encountered in achieving complex structures, especially with non‐native substrates. One possibility is that due to tight crystal packing, the substrates may be too bulky to reach the binding site. Alternatively, the otherwise flexible active site may rigidify in the crystal, preventing the ligand from binding. For smaller substrates such as tryptamine or indole derivatives, weaker binding may occur due to fewer and less specific interactions, thus preventing observation in the crystal. Additionally, the substrate may bind in multiple poses, resulting in multiple possible low‐occupancy binding poses that cannot be resolved unambiguously. This is the case, for example, for Thal‐RebH5, a Thal variant with altered regioselectivity, and the natural substrate L‐Trp.^[^ ^21^ ^]^ Docking of non‐native substrates is often ambiguous and usually generates several different binding poses.^[^ ^22^, ^36^, ^37^ ^]^

So far, only a few FDH structures with non‐native substrates have been published. We are aware of only four experimental FDH structures with non‐native substrates, namely, the RebH variant 0S with tryptamine^[^ ^22^ ^]^ (PBD 7ju0), Thal with d‐Trp (PDB: 8ad7) or a D‐Trp‐Ser dipeptide^[^ ^11^ ^]^ (PDB: 8ad8), and AetF with 7‐bromotryptophan^[^ ^38^ ^]^ (PDB: 8cjg). Our structure of Thal in complex with tryptoline continues this list and provides useful information to further alter the substrate binding site of halogenases to extend the repertoire of available biocatalysts.

## 3 Conclusion

In this study, we have demonstrated that Thal is able to halogenate a larger substrate more effectively than wild‐type RebH, presumably due to its more spacious active site. The crystal structure of Thal with tryptoline, a non‐native substrate, provides a useful lead structure for site‐directed mutagenesis of Thal to expand its substrate scope further. Since the same position is halogenated by Thal and its variants and by the RebH variants, and as the halogenation site differs from that of the native substrate, we showed that the regioselectivity of the native substrate cannot be transferred directly to structurally related non‐native substrates as observed before.^[^ ^10^, ^23^, ^39^, ^40^ ^]^ Since the binding pose of the pharmacophore shared between the native and the non‐native substrate differs, and because mutagenesis is likely to alter the active site, regioselectivity for the native substrate should not be the primary motivation for choosing a starting enzyme for the engineering campaign.

## 4 Experimental Section

4.1

4.1.1

##### Analytics

Reversed phase‐high performance liquid chromatography (RP‐HPLC) and high‐performance liquid chromatography‐mass spectrometry (LC‐MS) were done as described.^[^ ^41^ ^]^ NMR‐spectra were recorded as described.^[^ ^11^ ^]^

To determine the kinetics of Thal, different gradients were used for RP‐HPLC measurements. Tryptophan: (A %): 0–0.1 min: linear gradient from 95% to 85%, 0.1–6 min: 85%, 6–6.1 min: gradient from 85% to 2%, 6.1–8.0 min: 2%, 8.0–8.1 min: gradient from 2% to 95%, 8.1–10.0 min: 95%. Tryptoline: (A%): 0–0.1 min: linear gradient from 95% to 75%, 0.1–5.1 min: 75%, 5.1–6.0 min: gradient from 75% to 5%, 6.0–7.0 min: 5%, 7.0–7.1 min: gradient from 5% to 95%, 7.0–7.1 min: 95%.

##### Site‐Directed Mutagenesis

The Thal and RebH variants were prepared according to the standard procedure as described^[^ ^41^ ^]^ following the QuickChange II Site directed mutagenesis kit. Primers (Table S1, Supporting Information) were designed with the desired mismatches to incorporate the corresponding mutations.

##### Heterologous Expression in E. coli: Flavin Reductase PrnF and Halogenases Thal, RebH, and their Variants

50 mL of lysogeny broth (LB) medium containing kanamycin (60 μg mL^−1^) and chloramphenicol (50 μg mL^−1^) was inoculated with a glycerol culture of *E. coli* BL21 (DE3) pGro7 transformed with pET28a‐*prnF*, pET28a‐*thal*, pET28a‐*rebH*, or their variants and incubated overnight at 37 °C (150 rpm). 1.5 L of LB medium or 500 mL of TB medium containing kanamycin (60 μg mL^−1^) and chloramphenicol (50 g mL^−1^) was inoculated with 20 mL L^−1^ of the overnight culture. The culture was incubated at 37 °C (120 rpm) until an optical density at 600 nm (OD_600_) of 0.5–0.6 was reached. The culture was cooled down to 25 °C for 30 min, and protein expression was induced by the addition of 0.1 mM isopropyl‐β‐D‐1‐thiogalactopyranoside (IPTG) and 2 g L^−1^ L‐arabinose. After shaking for 20 h at 25 °C, cells were harvested by centrifugation (4,000 × g, 30 min, 4 °C), washed with Na_2_HPO_4_ buffer (40 mL, 0.1 M, pH 7.4), and stored at −20 °C.

##### Heterologous Expression in E. coli: PTDH

50 mL of LB medium containing kanamycin (60 μg mL^−1^) was inoculated with a glycerol culture of *E. coli* BL21 (DE3) transformed with pET28a‐*ptdh* and incubated overnight at 37 °C (150 rpm). 1.5 L of LB medium containing kanamycin (60 μg mL^−1^) was inoculated with 20 mL L^−1^ of the overnight culture. The culture was incubated at 37 °C (120 rpm) until an OD_600_ = 0.5–0.6 was reached. The culture was cooled down to 25 °C for 30 min, and protein expression was induced by addition of 0.1 mM IPTG. After shaking for 20 h at 25 °C, cells were harvested by centrifugation (4,000 × g, 30 min, 4 °C), washed with Na_2_HPO_4_ buffer (40 mL of 0.1 M, pH 7.4), and stored at −20 °C.

##### Heterologous Expression in E. coli: Protein Purification

His6‐tagged fusion protein was purified according to the standard procedure as described.^[^ ^41^ ^]^ For halogenases, the concentration was determined by Nano‐Drop UV spectroscopy with Abs 0.1% Thal = 1.410 and RebH = 1.452. The enzyme was dialyzed against 15 mM Na_2_HPO_4_ and 30 mM NaBr (pH 7.4, 5 L reservoir) overnight to remove the imidazole and chloride ions. Sodium dodecyl sulfate–polyacrylamide gel electrophoresis (SDS‐PAGEs) of purified Thal and RebH are shown in Figure S21, Supporting Information.

##### Heterologous Expression in E. coli: Determination of Flavin Reductase Activity

The volumetric activity of the flavin reductase PrnF was determined as triplicates by monitoring the decrease of absorption at 340 nm due to the oxidation of NADH to NAD+ (*ε* = 6.3 mL μmol^−1^ cm^−1^). The reaction was performed in a final volume of 1 mL containing 10 mM Na_2_HPO_4_ pH 7.4, 50 μM FAD, 160 μM NADH, and 20 μL of diluted flavin reductase (PrnF 1:1000). The conversion rate was determined using a UV‐3100 PC spectrometer (VWR) by regression of the linear range (15 s after addition of the enzyme).

##### Heterologous Expression in E. coli: Determination of PTDH Activity

The volumetric activity of PTDH was determined as triplicates by monitoring the increase of absorption at 340 nm due to the reduction of NAD^+^ to NADH (*ε* = 6.3 mL μmol^−1^ cm^−1^). The reaction was performed in a final volume of 1 mL containing 400 mM Na_2_HPO_3_ pH 7.4, 10 mM NAD^+^, and 10 μL of diluted PTDH (1:10). The conversion rate was determined using a UV‐3100 PC spectrometer (VWR) by regression of the linear range (15 s after addition of the enzyme).

##### Heterologous Expression in E. coli: Conversion of Tryptoline

The reaction mixture (final volume 250–700 μL) containing 10 μM halogenase, 1 mM tryptoline, 2.5 U mL^−1^ PrnF, 2 U mL^−1^ PTDH, 10 μM FAD, 1 mM NAD^+^, 50 mM Na_2_HPO_3_, 10 mM K_2_HPO_4_ pH 7.4, 30 mM NaBr, and dimethyl sulfoxide (DMSO) (5%) was incubated at 25 °C and 1050 rpm for 24–30 h. The reaction was quenched by addition of 1:1 methanol to the solution. Samples were centrifuged (30 min, 4,000 × g), diluted with dH_2_0 (1:1), and then analyzed using RP‐HPLC. The reaction was performed three times.

##### Heterologous Expression in E. coli: Determining the Kinetic Parameters of Thal for Bromination of Tryptophan and Tryptoline

The reaction mixture containing 10–1000 μM tryptoline and 5% DMSO or 5–500 μM tryptophan, 100 μM phenol, 2.5 U mL^−1^ PrnF, 2 U mL^−1^ PTDH, 10 μM FAD, 1 mM NAD^+^, 50 mM Na_2_HPO_3_, 10 mM K_2_HPO_4_ pH 7.4, and 30 mM NaBr was preincubated for 10 min before the reaction was started by the addition of 0.5 μM or 7 μM Thal and incubated at 25 °C and 500 rpm. The final reaction volume was 70 μL. The reaction was quenched with 100 μL of methanol after 5–30 min and flash cooled in liquid nitrogen until the samples were centrifuged and the supernatant was analyzed via RP‐HPLC. In order to determine the concentration, the quotient of the integral of the brominated product and phenol were compared to a calibration curve with known concentrations. The initial velocities were analyzed with a linear regression for the first 20 or 30 min. The kinetic parameters were analyzed using the Michaelis–Menten equation.

##### Conversion of Tryptoline in a Preparative Scale: Bromination of Tryptoline in a Preparative Scale with Cross‐Linked Enzyme Aggregates (CombiCLEAs)

For the immobilization, combiCLEAs were prepared as described.^[^ ^42^ ^]^ To regenerate NADH, 2 U mL^−1^ PTDH was added instead of ADH. The solid biocatalyst was resuspended in the reaction solution (1 L) containing 0.5 mM tryptoline, 1 μM FAD, 0.1 mM NAD^+^, 50 mM Na_2_HPO_3_, 15 mM Na_2_HPO_4_ pH 7.4, 30 mM NaBr, and DMSO (0.5%) and was incubated for 2 days. To isolate the bromotryptoline, the solid catalyst was removed by filtration, and the solution was concentrated under reduced pressure and then extracted three times with dichloromethane (DCM). After DCM had been removed, the product was purified by preparative RP‐HPLC (Hypersil GOLD Preparative HPLC Column (250 nm × 10 mm ID, 8.0 μm), 0–5 min 5% acetonitrile/ 95% water 0.1% trifluoroacetic acid (TFA), 5–105 min 5% acetonitrile/95% water/0.1% TFA to 95% acetonitrile/5% water/0.1% TFA). The TFA salt of bromotryptoline was isolated in a yield of 15.6% (28 mg, 0.079 mmol, 6‐bromotryptoline (≈85%)/7‐bromotryptoline (≈15%)).

##### Conversion of Tryptoline in a Preparative Scale: Regioselectivity of Thal G113S and Thal G469S

To analyze the regioselectivity of Thal G113S and Thal G469S, the reaction was performed as described above (conversion of tryptoline) but with a final volume of 15 to 21 mL. For purification, the enzymes were denatured after 30 h by adding 1:1 acetonitrile to the reaction mixture. The precipitated enzymes were removed by centrifugation. The solution was lyophilized, and the crude product was dissolved in water/acetonitrile (1:1). Bromotryptoline was purified by preparative RP‐HPLC (Luna (5 μm C18(2) 100 Å), 0–10 min 5% acetonitrile/ 95% water 0.1 % TFA, 10–70 min 5 to 60% acetonitrile/ 95 to 40% water 0.1% TFA). Fractions containing the product were combined and lyophilized. The ratio of the regioisomers was determined using the integrated peak areas of the C5H (6‐bromotryptoline) and the C8H (7‐bromotryptoline) peaks in the ^1^ H‐NMR spectra.

##### Conversion of Tryptoline in a Preparative Scale: Regioselectivity of RebH N470S and RebH 2S

To analyze the regioselectivity of RebH N470S and RebH 2S, the reaction was performed as described above (conversion of tryptoline) but with 10 mL of halogenase lysate instead of purified enzyme and a final volume of 50 mL. For purification, the enzymes were precipitated and tryptoline/bromotryptoline was isolated after 44 h by extraction with DCM. The crude product was purified by preparative RP‐HPLC and analyzed as described above (regioselectivity of Thal G113S and Thal G469S).

##### Conversion of Tryptoline in a Preparative Scale: Expression, Purification, and Crystallization of Thal in Complex with Tryptoline

Thal was expressed and purified as described,^[^ ^21^ ^]^ except for using Co‐NTA (Cube Biotech) instead of Ni–NTA. Thal was diluted to 15 mg mL^−1^ with a crystallization buffer (10 mM Tris pH 7.4, 50 mM NaCl, 1 mM dithiothreitol (DTT)) and crystallized in MRC 2‐lens sitting‐drop plates (SWISSCI) with a ratio of 100 nL protein solution mixed with a 100 nL reservoir solution (0.1 M bicine pH 8.0, 1.6 M KH_2_PO_4_/K_2_HPO_4_). Within 7 days, crystals appeared as hexagonal prisms and were soaked with tryptoline by adding 100 nL of soaking solution (saturated tryptoline in 0.1 M bicine pH 8.5, 1.5 M KH_2_PO_4_/K_2_HPO_4_) to the crystallization drop for 60 min. Crystals were transferred into cryoprotection buffer (400 nL of soaking solution + 300 nL of reservoir solution + 300 nL of glycerol) before flash‐cooling in liquid nitrogen.

##### Conversion of Tryptoline in a Preparative Scale: Data Collection and Structure Determination

X‐ray diffraction data were collected on beamline BL14.2 at the BESSY II electron storage ring operated by the Helmholtz‐Zentrum Berlin für Materialien und Energie.^[^ ^43^ ^]^ Diffraction data were processed with *XDS* and scaled with *XSCALE*.^[^ ^44^ ^]^ The structure was phased via the difference Fourier method by refining an apo version of Trp:Thal (PDB: 6h44) as a rigid body against our data in *phenix.refine*.^[^ ^45^, ^46^ ^]^ The model was improved by several rounds of model building in *Coot* ^[^ ^47^ ^]^ and refinement utilizing noncrystallographic symmetry restraints and refining occupancies of all placed ligands in *phenix.refine*. The structure and restraints for tryptoline (PDB ligand ID: WYH) were generated in *eLBOW* using the AM1 QM method.^[^ ^48^ ^]^ The resolution limit was determined with paired refinement^[^ ^49^ ^]^ as implemented in *PAIREF*.^[^ ^50^ ^]^ Data collection and refinement statistics are summarized in Table S2, Supporting Information.^[^ ^29^ ^]^All structural figures were generated using *PyMOL*. The coordinates and structure factors of tryptoline:Thal were deposited in the Protein Data Bank with accession code 8rs4. Diffraction images are available via the SBGrid Data Bank.^[^ ^51^ ^]^

##### Conversion of Tryptoline in a Preparative Scale: Replacement of Tryptoline by Bromo‐Tryptoline In Silico

The structure and restraints for potential halogenation products were generated in *eLBOW* using the AM1 QM method.^[^ ^48^ ^]^ To replace tryptoline by potential halogenation products, the *Guided Ligand Replacement* module^[^ ^29^ ^]^ was used. This finds the best structural match of brominated tryptoline to the non‐brominated tryptoline of the experimental structure.

## Conflict of Interest

The authors declare no conflict of interest.

## Supporting information

Supplementary Material

## Data Availability

The coordinates and structure factors of tryptoline:Thal were deposited in the Protein Data Bank with accession code 8rs4. Diffraction images are available via the SBGrid Data Bank.
