# Supplementary material for: Structural Basis of Regioselective Bromination of Tricyclic Tryptoline by the Tryptophan Halogenase Thal
Source: Chembiochem. 2025 Jun 17;26(13):e202500246. doi: 10.1002/cbic.202500246 (PMC12247023; doi:10.1002/cbic.202500246)
Supplement: Supplementary file 1 — Supplementary Material [file CBIC-26-e202500246-s001.pdf]

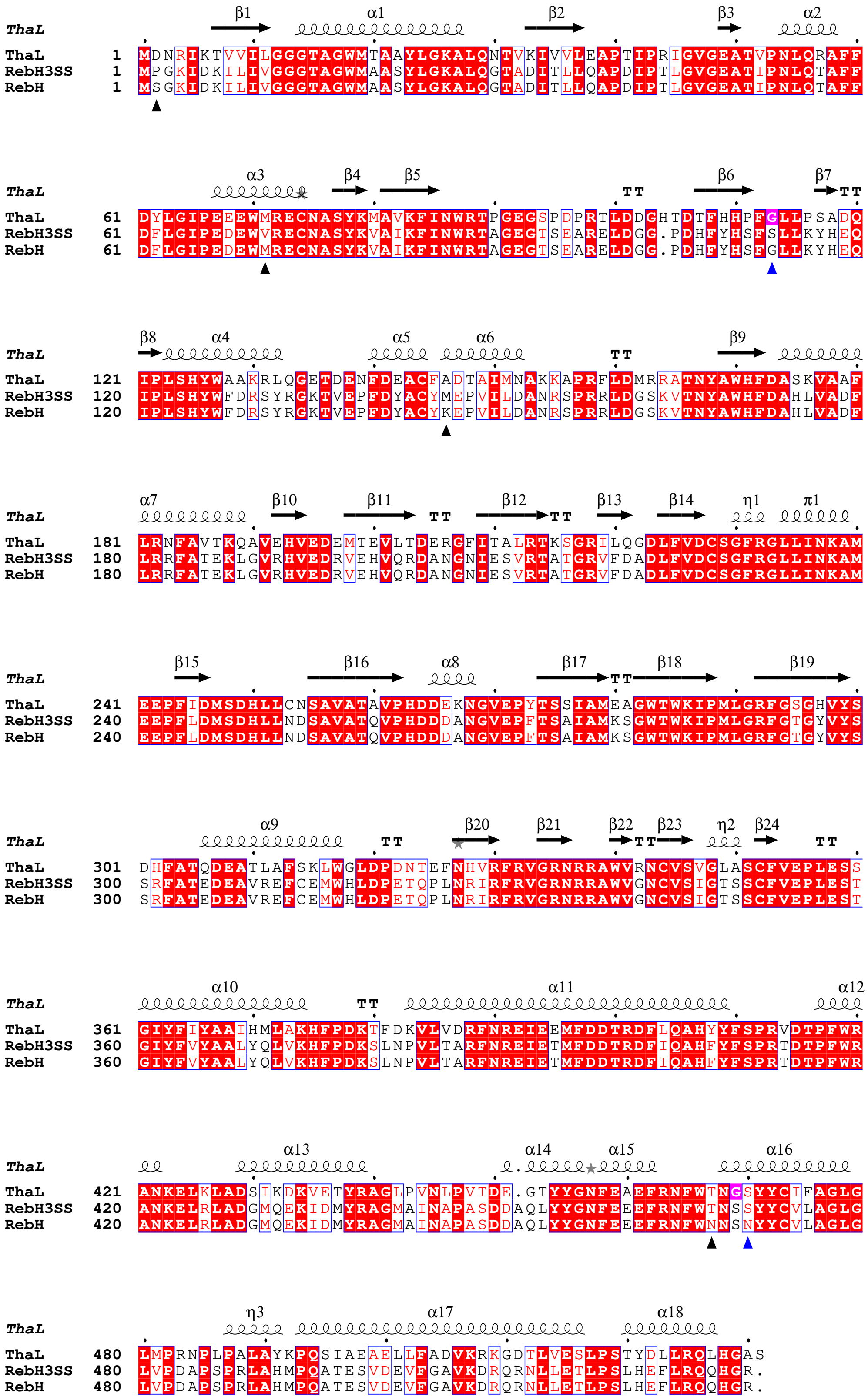

Figure S1. Sequence alignement (Clustal Omega) of Thal, RebH3SS and RebH. Important residues in RebH identified via directed evolution done by the Lewis group[10] are marked with black arrows. Mutations of the last evolution step resulting in the RebH3SS variant are marked with blue arrows. Based on RebH3SS mutations in Thal (G113S and G469S) were selected in this study (residues shown in magenta).

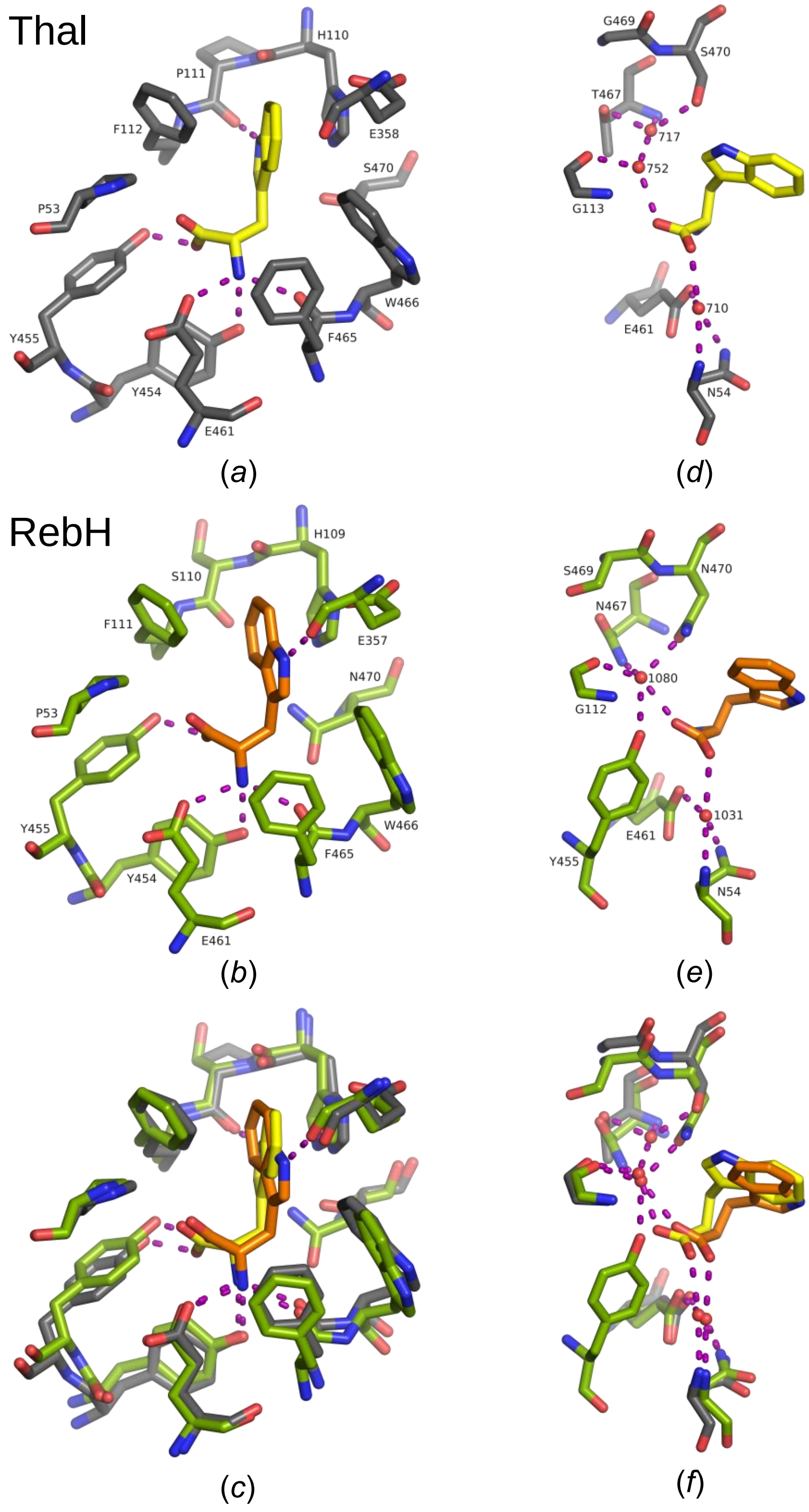

Thal	RebH 3SS	RebH wt
P53	P53	P53
N54	N54	N54
H110	H109	H109
P111	S110	S110
F112	F111	F111
G113	S112	G112
E358	E358	E358
Y454	Y454	Y454
Y455	Y455	Y455
E461	E461	E461
F465	F465	F465
W466	W466	W466
T467	T467	N467
G469	S469	S469
S470	S470	N470

(*g*)

Figure S2. Structure and sequence comparison of the substrate binding site in Thal, RebH and RebH 3SS (sequence only).
(a-c) Stick models of equivalent residues from both Thal and RebH if they have a distance of 3.5 Å or less to the substrate Trp in either Thal or RebH. Polar contacts are shown as purple dotted lines. (a) Thal (PDB 6h44) with carbon atoms of the protein in gray and carbon atoms of the substrate Trp in yellow. (b) RebH (PDB 2e4g) with carbon atoms of the protein in green and carbon atoms of the substrate Trp in orange. (c) Overlay of Thal and RebH.
(d-f) Stick models of residues forming hydrogen bonds to water molecules that are directly bound to the substrate Trp and residue 469 that differs between Thal (Gly) and RebH (Ser). The coloring is the same as in panels a-c. (d) Thal. (e) RebH. (f) Overlay of Thal and RebH.
(g) Sequence comparison of Thal, RebH and RebH 3SS. Note that the numbering of Thal and RebH differs by one for residues 110-113 (Thal). Residues that are identical in two or more proteins are shown in green. Residues that differ from the other two proteins are shown in yellow. Note that the side chain of Pro111 (Thal) / Ser110 (RebH) points away from the substrate, suggesting that this substitution might not influence substrate binding.

Analysis: Bromination of tryptoline by Thal WT

The TFA salt of 6- and 7-bromotryptoline was isolated in a yield of 15.6 % (28 mg, 0.079 mmol, (6-bromotryptoline (~85 %)/ 7-bromotryptoline (~15 %).

LC-MS: $[C_{11}H_{11}{}^{79}BrN_2+H]^+$ *m/z* = 251,02 (calc.: 251,02), $[C_{11}H_{11}{}^{81}BrN_2+H]^+$ *m/z* = 253,02 (calc.: 253,02) RT: 4.75 min.

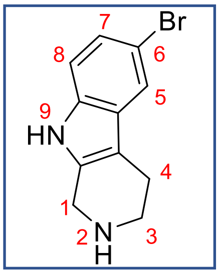

6-Bromo-2,3,4,9-tetrahydro-1*H*-pyrido[3,4-b]indole (6-bromotryptoline)

^{1}H NMR (600 MHz, DMSO-d6) δ 11.31 (s, 1H, N^9**H**), 9.31 (s, 1H, N^2**H**), 7.68 (d, ^4J = 1.8 Hz, 1H, C^5**H**), 7.35 (d, ^3J = 8.6 Hz, 1H, C^8**H**), 7.22 (dd, J^3 = 8.6, ^4J = 1.9 Hz, 1H, C^7**H**), 4.37 (s, 2H, C^1**H$_2$**), 3.45 (t, ^3J = 5.8 Hz, 2H, C^3**H$_2$**), 2.92 (t, ^3J = 5.9 Hz, 2H, C^4**H$_2$**)
^{13}C NMR (600 MHz, DMSO-d6) δ 135.2 (C^{8a}), 128.9 (C^{9a}), 128.2 (C^{4b}), 124.5 (C^7), 120.8 (C^5), 113.8 (C^8), 112.0 (C^6), 105.8 (C^{4a}), 41.9 (C^3), 40.8 (C^1), 18.4 (C^4)

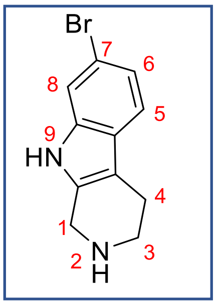

7-Bromo-2,3,4,9-tetrahydro-1*H*-pyrido[3,4-b]indole (7-bromotryptoline)

^{1}H NMR (600 MHz, DMSO-d6) δ 11.33 (s, 1H, N^9**H**), 9.31 (s, 1H, N^2**H**), 7.59 (d, ^4J= 1.7 Hz, 1H, C^8**H**), 7.43 (d, ^3J = 8.38 Hz, 1H, C^5**H**), 7.15 (dd, ^3J = 8.4, ^4J = 1.8 Hz, 1H, C^6**H**), 4.36 (s, 2H, C^1**H$_2$**), 3.45 (t, ^3J = 5.8 Hz, 2H, C^3**H$_2$**), 2.92 (t, ^3J = 5.9 Hz, 2H, C^4**H$_2$**)
^{13}C NMR (600 MHz, DMSO-d6) δ 137.4 (C^{8a}), 128.3 (C^{9a}), 125.4 (C^{4b}), 122.3 (C^6), 120.1 (C^5), 114.7 (C^7), 114.5 (C^8), 106.3 (C^{4a}), 41.9 (C^3), 40.8 (C^1), 18.4 (C^4)

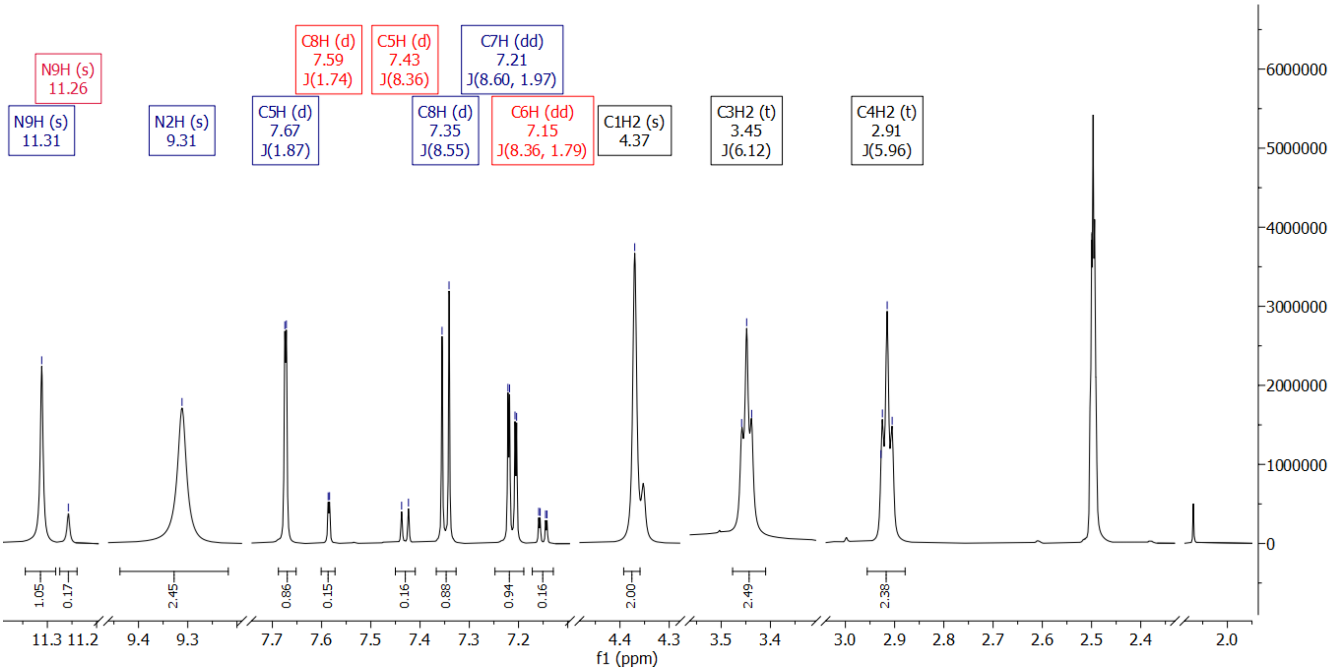

Figure S3. ^{1}H-NMR (600 MHz, DMSO-d6) spectrum of 6-bromotryptoline (blue) and 7-bromotryptoline (red) synthesized with Thal

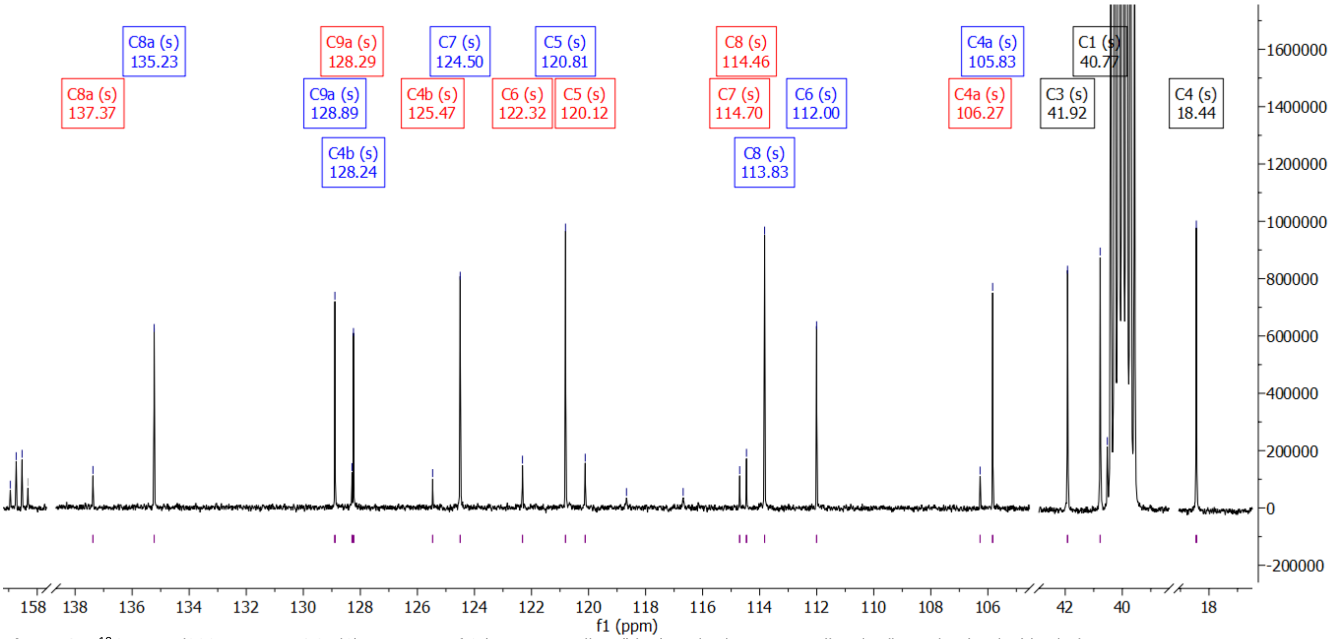

Figure S4: ^{13}C-NMR (600 MHz, DMSO-d6) spectrum of 6-bromotryptoline (blue) and 7-bromotryptoline (red) synthesized with Thal WT

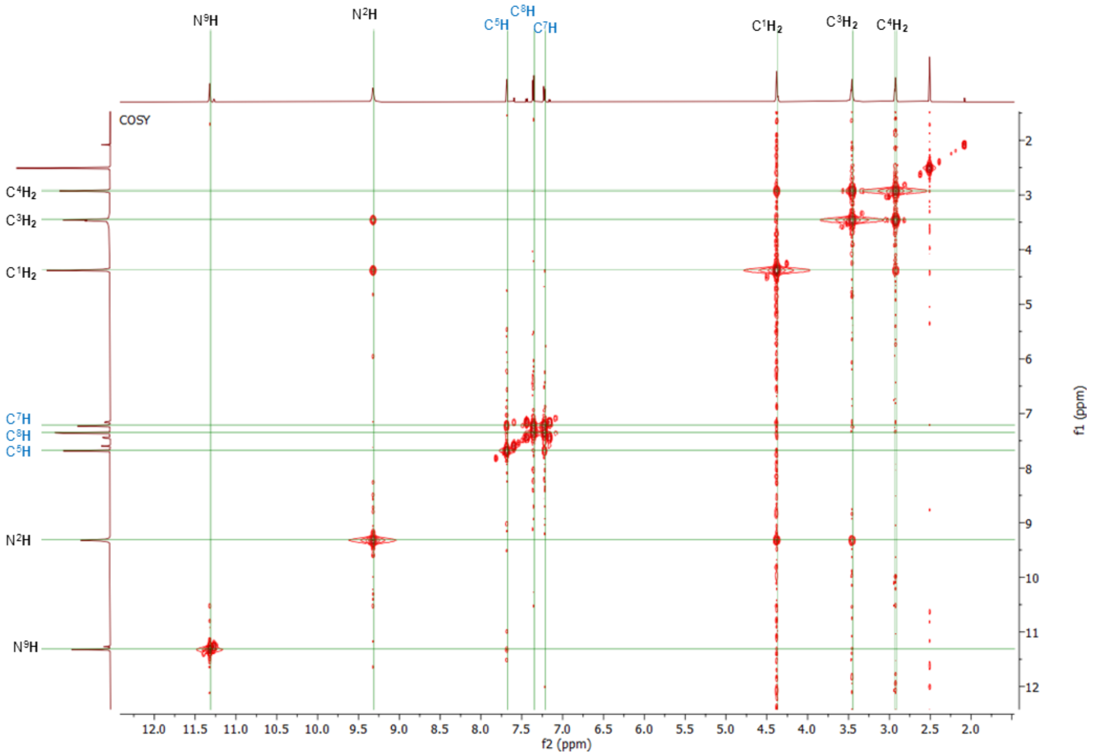

Figure S5. COSY (600 MHz, DMSO-d6) spectrum of 6-bromotryptoline (blue) and 7-bromotryptoline synthesized with Thal WT

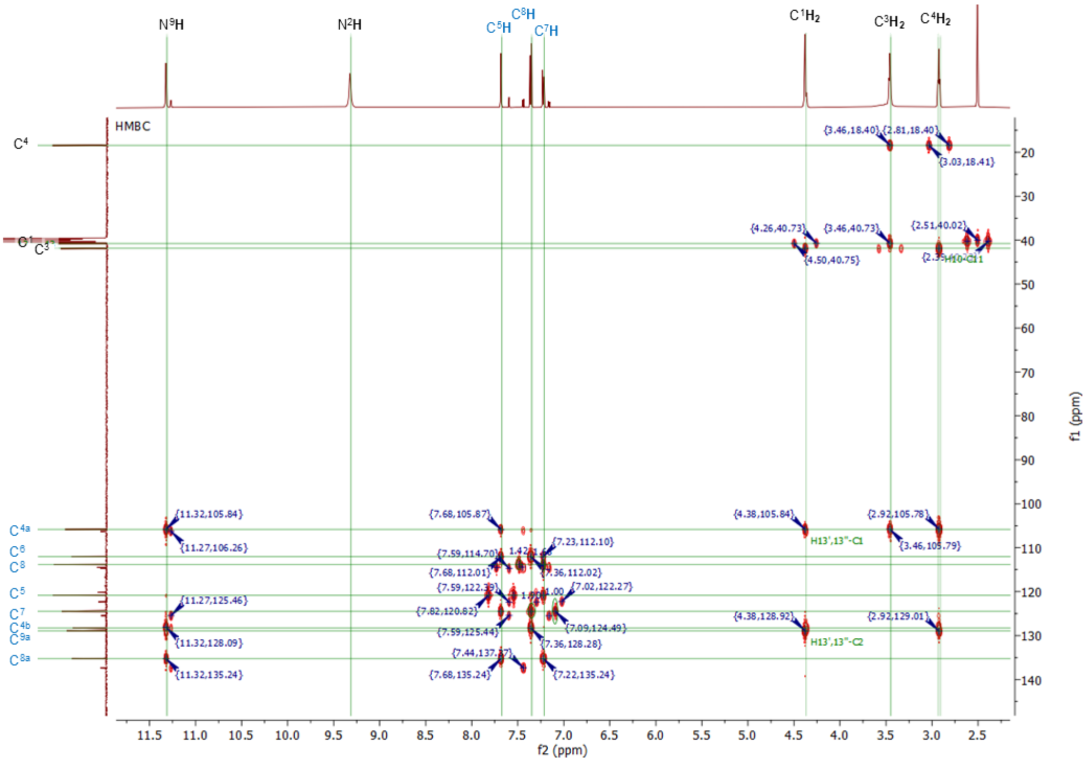

Figure S6. HMBC (600 MHz, DMSO-d6) spectrum of 6-bromotryptoline (blue) and 7-bromotryptoline synthesized with Thal WT

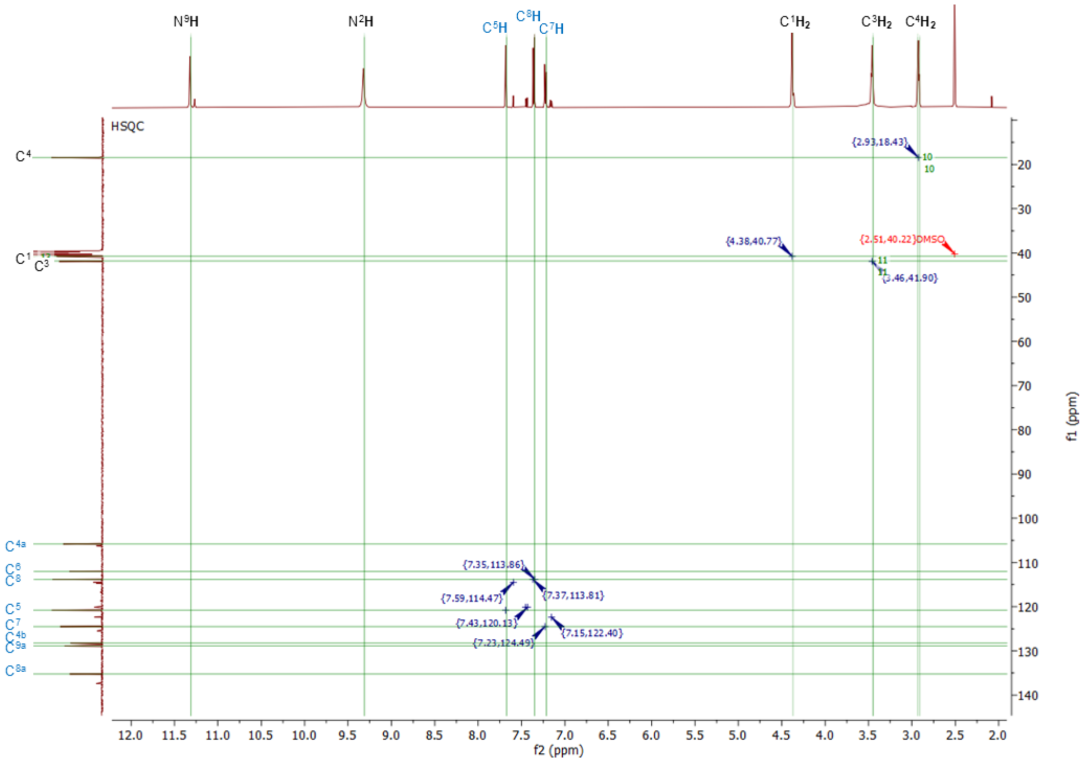

Figure S7. HSQC (600 MHz, DMSO-d6) spectrum of 6-bromotryptoline and 7-bromotryptoline synthesized with Thal WT

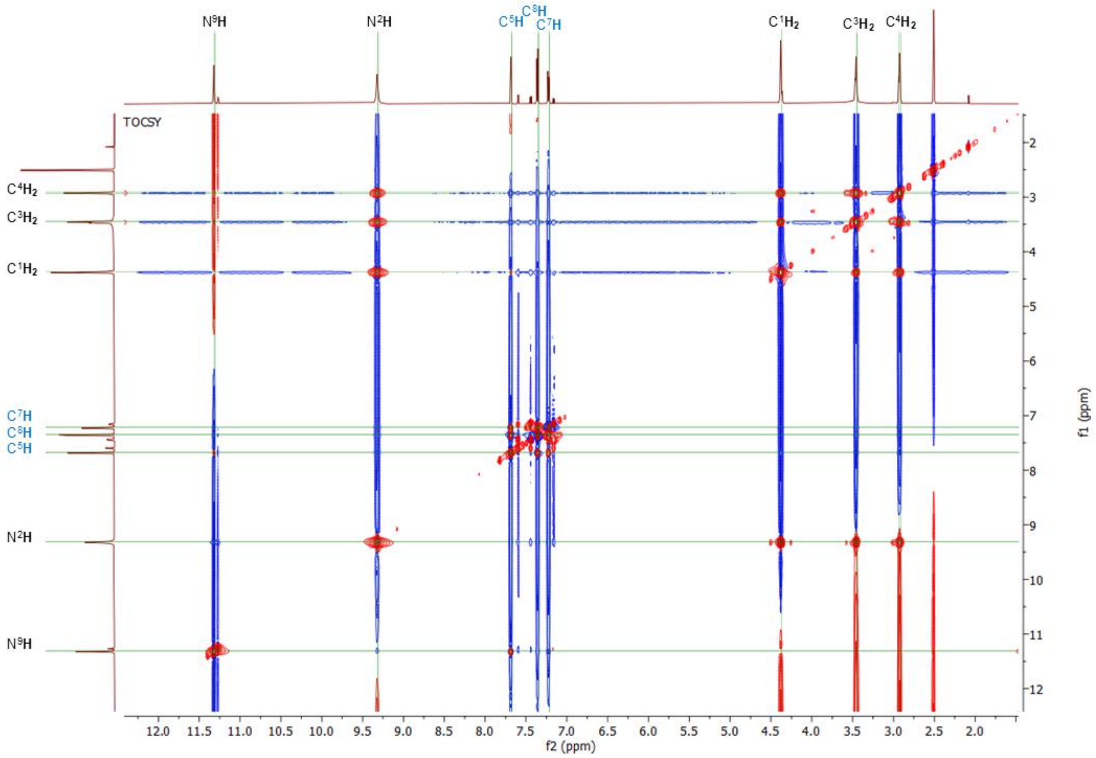

Figure S8. TOCSY (600 MHz, DMSO-d6) spectrum of 6-bromotryptoline and 7-bromotryptoline synthesized with Thal WT

Analysis: Bromination of tryptoline by Thal G113S

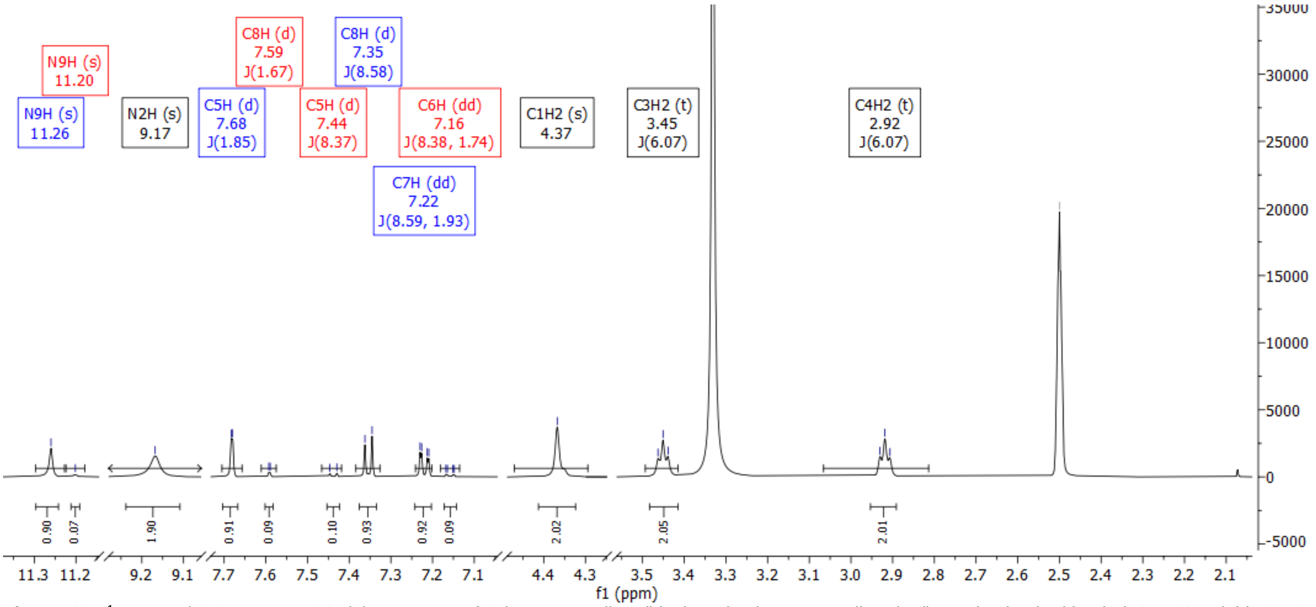

Figure S9. ^{1}H-NMR (600 MHz, DMSO-d6) spectrum of 6-bromotryptoline (blue) and 7-bromotryptoline (red) synthesized with Thal G113S. Yield: 20 % (6-bromotryptoline (~91 %)/ 7-bromotryptoline (~9 %))

Analysis: Bromination of tryptoline by Thal G469S

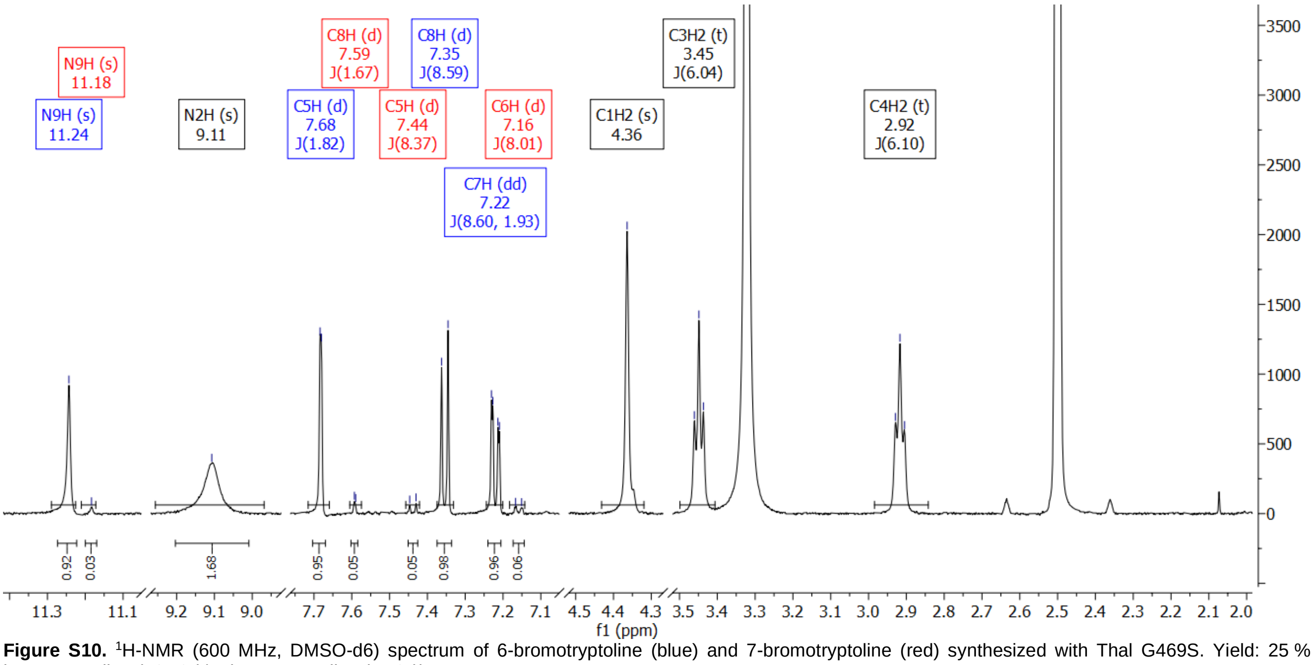

Figure S10. ^{1}H-NMR (600 MHz, DMSO-d6) spectrum of 6-bromotryptoline (blue) and 7-bromotryptoline (red) synthesized with Thal G469S. Yield: 25 % (6-bromotryptoline (~95 %)/ 7-bromotryptoline (~5 %))

Analysis: Bromination of tryptoline by RebH N470S

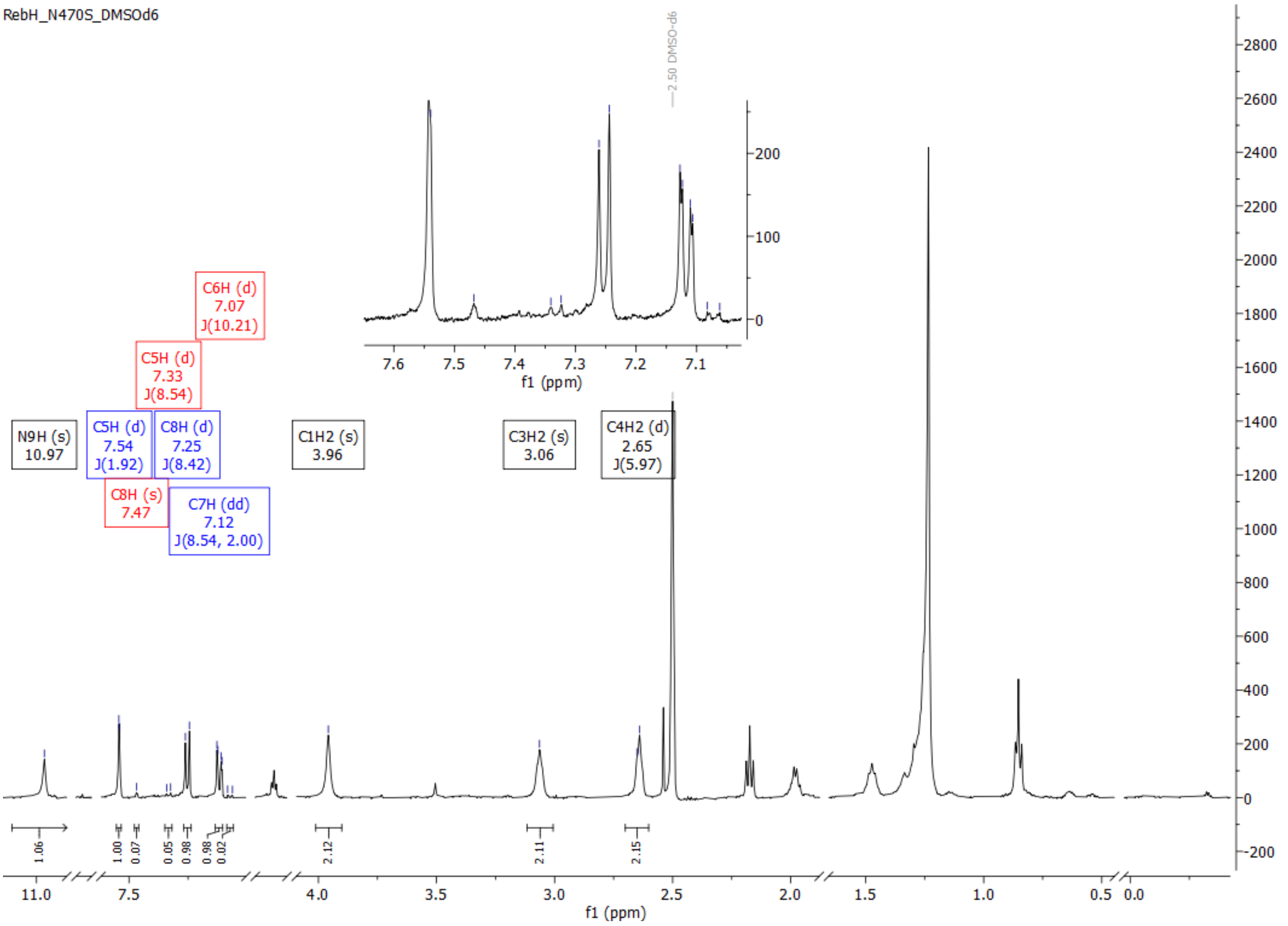

Figure S11. ^{1}H-NMR (500 MHz, DMSO-d6) spectrum of 6-bromotryptoline (blue) and 7-bromotryptoline (red) synthesized with RebH N470S. Yield: 25 % (6-bromotryptoline (~95 %)/ 7-bromotryptoline (~5 %))

Analysis: Bromination of tryptoline by RebH 2S

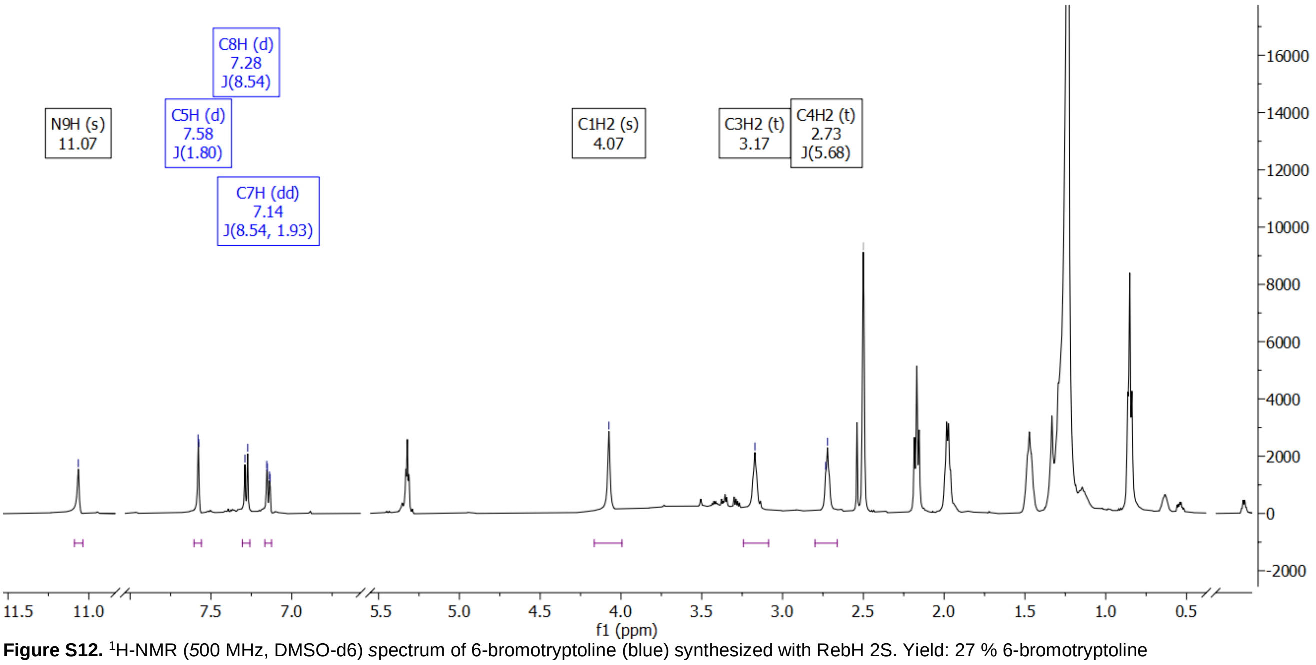

Figure S12. ^{1}H-NMR (500 MHz, DMSO-d6) spectrum of 6-bromotryptoline (blue) synthesized with RebH 2S. Yield: 27 % 6-bromotryptoline

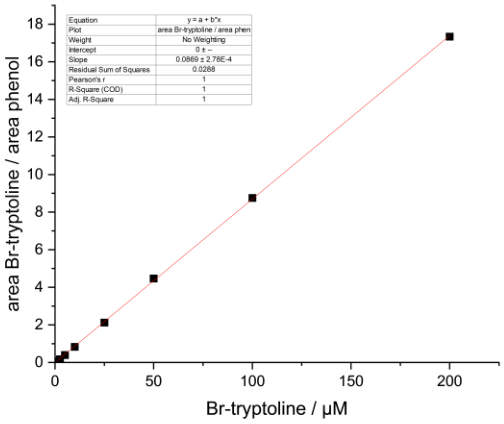

Figure S13. Calibration curve for calculating the concentration of bromotryptoline in the kinetics measurement.

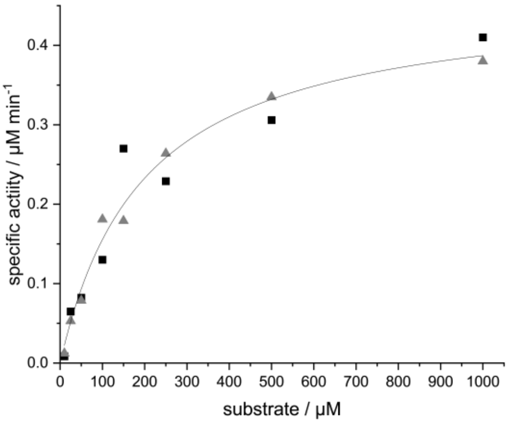

Figure S14. Michaelis-Menten curve for Thal wild type (7µM) with tryptoline as substrate.

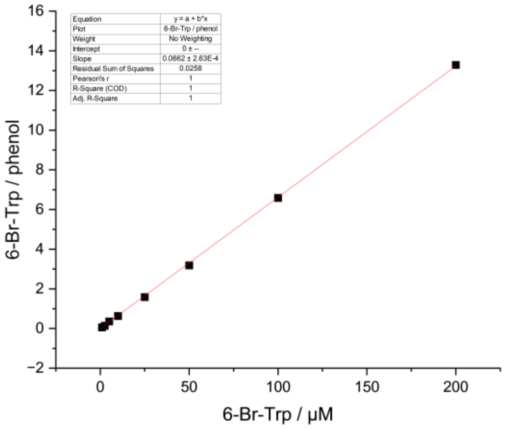

Figure S15. Calibration curve for calculating the concentration of 6-bromotryptophane in the kinetics measurement.

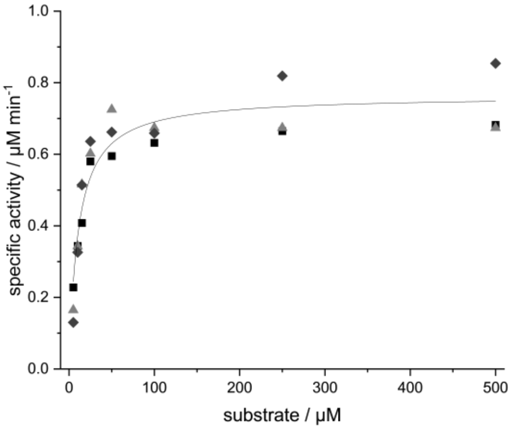

Figure S16. Michaelis-Menten curve for Thal wild type (0.5 µM) with L-tryptophan as substrate.

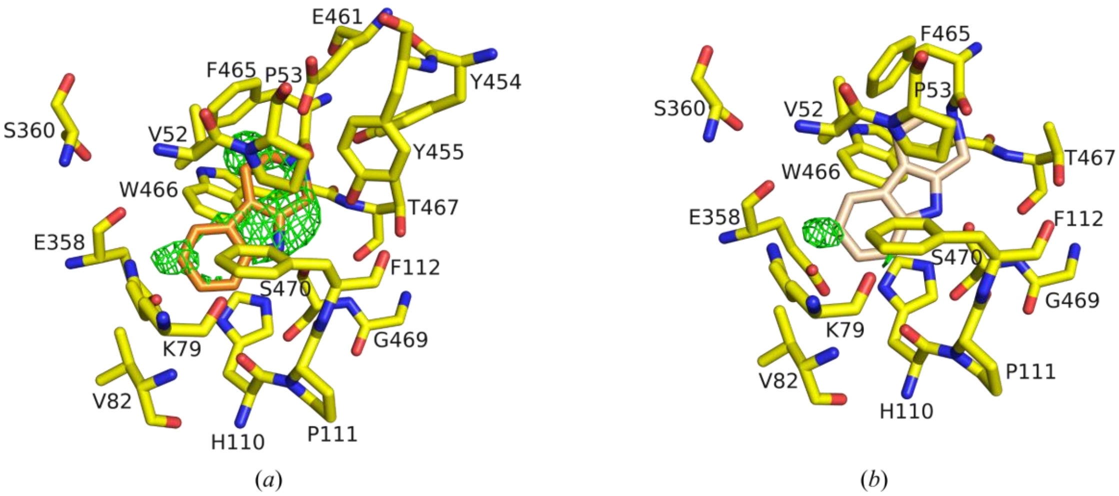

Figure S17. Active site of chain A (a) and chain B (b) with *Fo-Fc* density (green mesh, 3 σ) before placing tryptoline. Final position of tryptoline shown with orange carbon atoms for chain A. For chain B the tryptoline from chain A was copied via NCS and is shown with wheat carbon atoms.

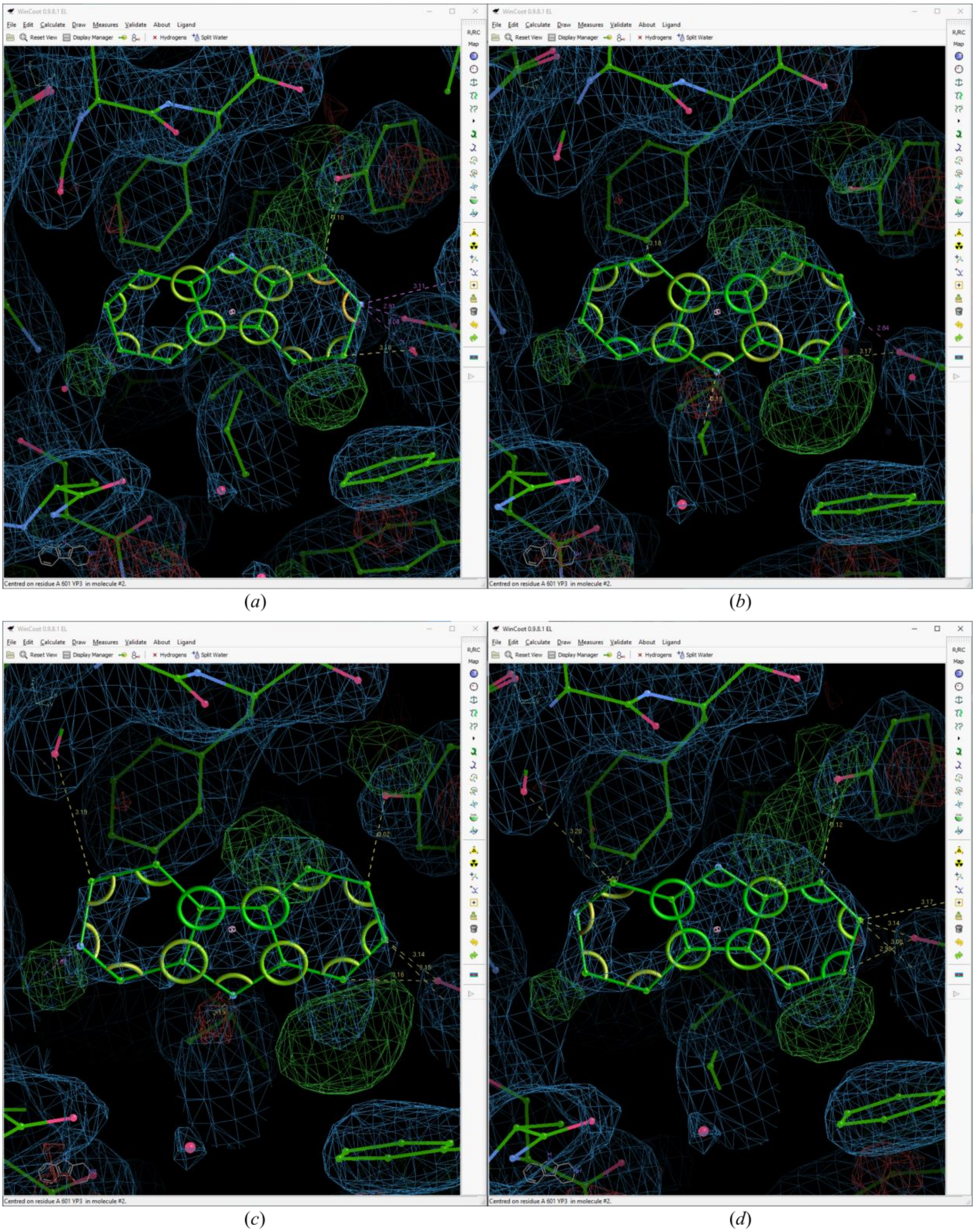

Figure S18. Tested binding poses of tryptoline in active center of chain A with displayed ligand distortions. Only binding pose a) results in a good explanation of the electron densitiy and reasonable contacts to surrounding amino acids.

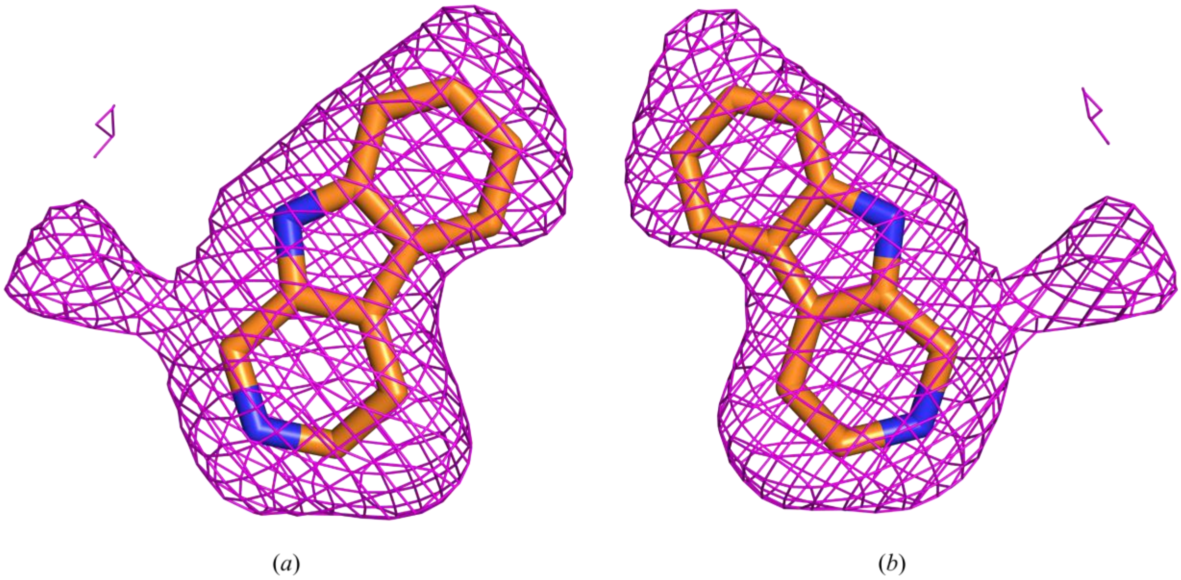

Figure S19. A polder map (magenta mesh, 3 σ) with tryptoline (orange carbon atoms) as omitted molecule reveals no alternative binding poses. Both views are rotated by 180°.

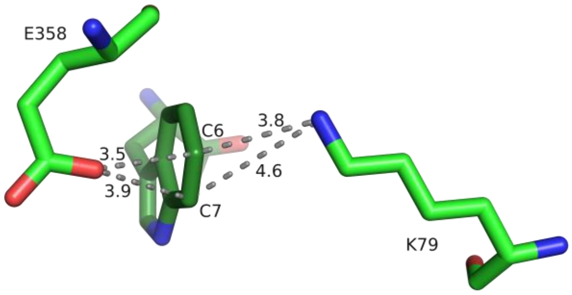

Figure S20. Comparison of the C6 and C7 distances (in Å) of L-Trp to K79 and E358 in Thal (PDB: 6h44).

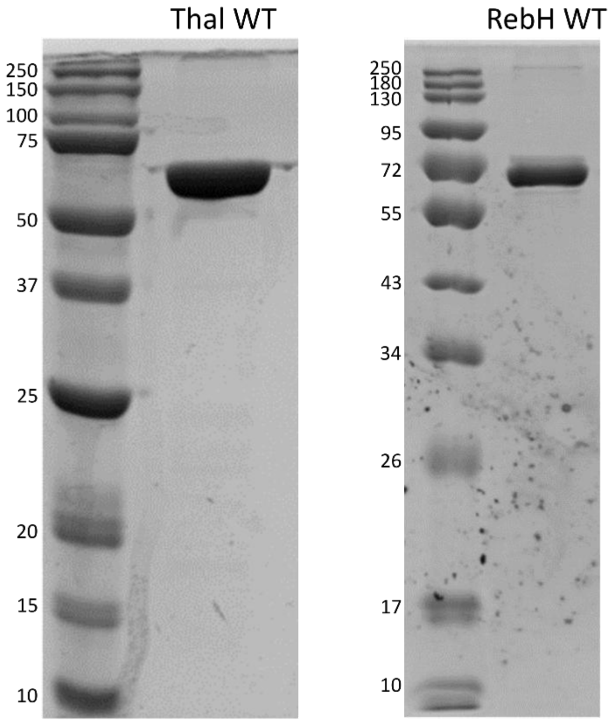

Figure S21. Non-reductive SDS-PAGEs of Thal and RebH used for catalytic assays. In both cases the visible band is in agreement with the expected molecular weight of ~60 kDa.

Table S1. List of primer sequences for the generation of Thal and RebH variants by site-directed mutagenesis where FW corresponds to the forward and RV to the reverse primer.

Primer[b]		Sequence 5´->3´
RebH_ G112S	FW	CTACCACTCCTTCAGCCTGCTCAAGTACC
	RV	GGTACTTGAGCAGGCTGAAGGAGTGGTAG
RebH_ N470S	FW	CTGGAACAACAGCAGCTACTACTGCGTGC
	RV	GCACGCAGTAGTAGCTGCTGTTGTTCCAG
Thal_ G113S	FW	CTTTCATCATCCGTTTAGCCTGCTGCCGAG
	RV	CTCGGCAGCAGGCTAAACGGATGATGAAAG
Thal_ G469S	FW	CTTTTGGACCAATAGCAGCTATTATTGTATCTTTGCC
	RV	GGCAAAGATACAATAATAGCTGCTATTGGTCCAAAAG

Table S2. Data collection and refinement statistics.

	Tryptoline:Thal
PDB ID	8rs4
DOI of raw data (images)	10.15785/SBGRID/1079
Wavelength (Å)	0.9184
Space group	$P6_4$
a, *b*, *c* (Å)	137.59, 137.59, 144.05
α, β, γ (°)	90, 90, 120
**Data collection statistics**	
Resolution range (Å)	50.0 – 2.20 (2.26 – 2.20)
Total No. of reflections	819088 (54883)
No. of unique reflections	78305 (5792)
Completeness (%)	100 (100)
Redundancy	10.46 (9.48)
$\langle I/\sigma(I)\rangle$	15.35 (0.99)
$CC_{1/2}$	0.998 (0.353)
R_{meas} (%)	15.7 (256.0)
Mosaicity (°)	0.118
Overall *B* from Wilson plot (Å^2)	42.80
**Refinement and model statistics**	
Resolution range (Å)	45.04 – 2.20 (2.23 – 2.20)
Completeness (%)	99.9 (100)
No. of reflections, working set	78242 (2658)
No. of reflections, test set	3913 (140)
Final R_{work} (%)	16.88 (28.90)
Final R_{free} (%)	20.37 (29.43)
No. of non-H atoms	9219
Protein	8533
Ligand	126
Water	560
R.m.s. deviations	
Bonds (Å)	0.008
Angles (°)	1.136
Average *B* factors (Å^2)	53.71
Protein	53.80
Ligand	60.01
Water	50.84
Ramachandran plot	
Most favoured (%)	98
Allowed (%)	2
Outliers (%)	0

References

[10] J. T. Payne, C. B. Poor, J. C. Lewis, *Angewandte Chemie - International Edition* **2015**, *54*, 4226–4230.
